# Nuclear translocation of SLC25A10 isoform 3 promotes chemoresistance in HCC cells via CEBPB/BCL2A1 signaling

**DOI:** 10.1038/s41419-026-08667-4

**Published:** 2026-04-09

**Authors:** Dan Liu, Shuang Dong, Siwei Cheng, Yuanyuan Lin, Sheng Chen, Jiaxin Gao, Bo Bi, Na Li, Jun Mi, Wujun Xiong

**Affiliations:** 1 Hongqiao International Institute of Medicine, Tongren Hospital; Basic Medical Institute; Key Laboratory of Cell Differentiation and Apoptosis of the Chinese Ministry of Education, Shanghai Jiao Tong University School of Medicine, Shanghai, China; 2 Department of Gastroenterology, Shanghai Pudong Hospital, Fudan University Pudong Medical Center, Fudan University Pudong Medical Center, Shanghai, China; 3 Institute of Cancer Stem Cell, Dalian Medical University, Dalian, China

**Keywords:** Liver cancer, Apoptosis

## Abstract

SLC25A10, the mitochondrial dicarboxylate carrier, plays a crucial role in mitochondrial metabolism and protects against liver lipotoxicity. Moreover, its frequent amplification or mutation in cancers, particularly hepatocellular carcinoma (HCC), correlates with a poor prognosis. This study aimed to investigate the role of SLC25A10 in chemotherapy resistance in HCC and elucidate the underlying mechanisms. In this study, we found that hypoxia increased SLC25A10 expression with a preferential shift toward isoform 3 in HCC. This isoform interacts with the nuclear transporter IPO7 to translocate into the nucleus, where it binds to transcription factor CEBPB. This interaction upregulates the transcription of the anti-apoptotic gene *BCL2A1*, thereby enhancing HCC cell resistance to the chemotherapeutic agent, etoposide. Notably, disruption of the SLC25A10 isoform 3-IPO7 interaction significantly sensitized HCC tumors to etoposide in vivo, suggesting that targeting this interaction could be a promising therapeutic strategy to improve chemotherapy efficacy in HCC. This study reveals a novel nuclear function of the mitochondrial dicarboxylate carrier SLC25A10 in transcriptional regulation under hypoxic conditions, distinct from its canonical mitochondrial role. These findings expand our understanding of SLC25A10 biology and uncover a previously unrecognized mechanism that drives hypoxia-induced chemoresistance in HCC. Our findings suggest that SLC25A10 is a potential therapeutic target to overcome drug resistance in HCC.

## Introduction

The solute carrier (SLC) family plays a pivotal role in the transport of glucose, amino acids, and a wide range of intermediate metabolites [1, 2]. Among its members, SLC25A10-also known as the mitochondrial dicarboxylate carrier, is localized in the inner mitochondrial membrane, where it mediates the exchange of malate and succinate [3]. These two metabolites are key intermediates in the tricarboxylic acid (TCA) cycle [4]. In addition to its role in the TCA cycle, SLC25A10 also supplies malate for citrate export, a critical step in fatty acid biosynthesis, and has been implicated in protecting against liver lipotoxicity induced by a high-fat diet [5, 6]. Collectively, these functions highlight the essential contribution of SLC25A10 to mitochondrial metabolism and its broad physiological significance within the SLC family.

Analysis of data from The Cancer Genome Atlas (TCGA; www.cancergenome.nih.gov) indicates that *SLC25A10* is frequently amplified or mutated across multiple tumor types, including breast cancer, mesothelioma, and hepatocellular carcinoma (HCC), with amplification rates exceeding 5% [7]. Gene expression profiling further revealed that *SLC25A10* is upregulated in HCC. Notably, elevated expression of SLC25A10 has been linked to regulates mitochondrial function and enhanced antioxidant capacity in osteosarcoma, lung and breast cancers, and is associated with poorer overall survival [8–10]. Collectively, these findings suggest that SLC25A10 plays a critical role in tumor progression, including the development and progression of HCC.

This study aimed to investigate the role of SLC25A10 in chemotherapy resistance in HCC and elucidate the underlying mechanisms. Our findings reveal that in addition to its canonical function as a mitochondrial dicarboxylate carrier, SLC25A10 also plays a regulatory role in transcription, contributing to its capacity to promote chemotherapy resistance in HCC. These insights expand our understanding of the functional versatility of SLC25A10 and highlight its potential as a therapeutic target for cancer treatment.

## Results

### The SLC25A10 plays a crucial role in HCC progression

**Fig. 1:**
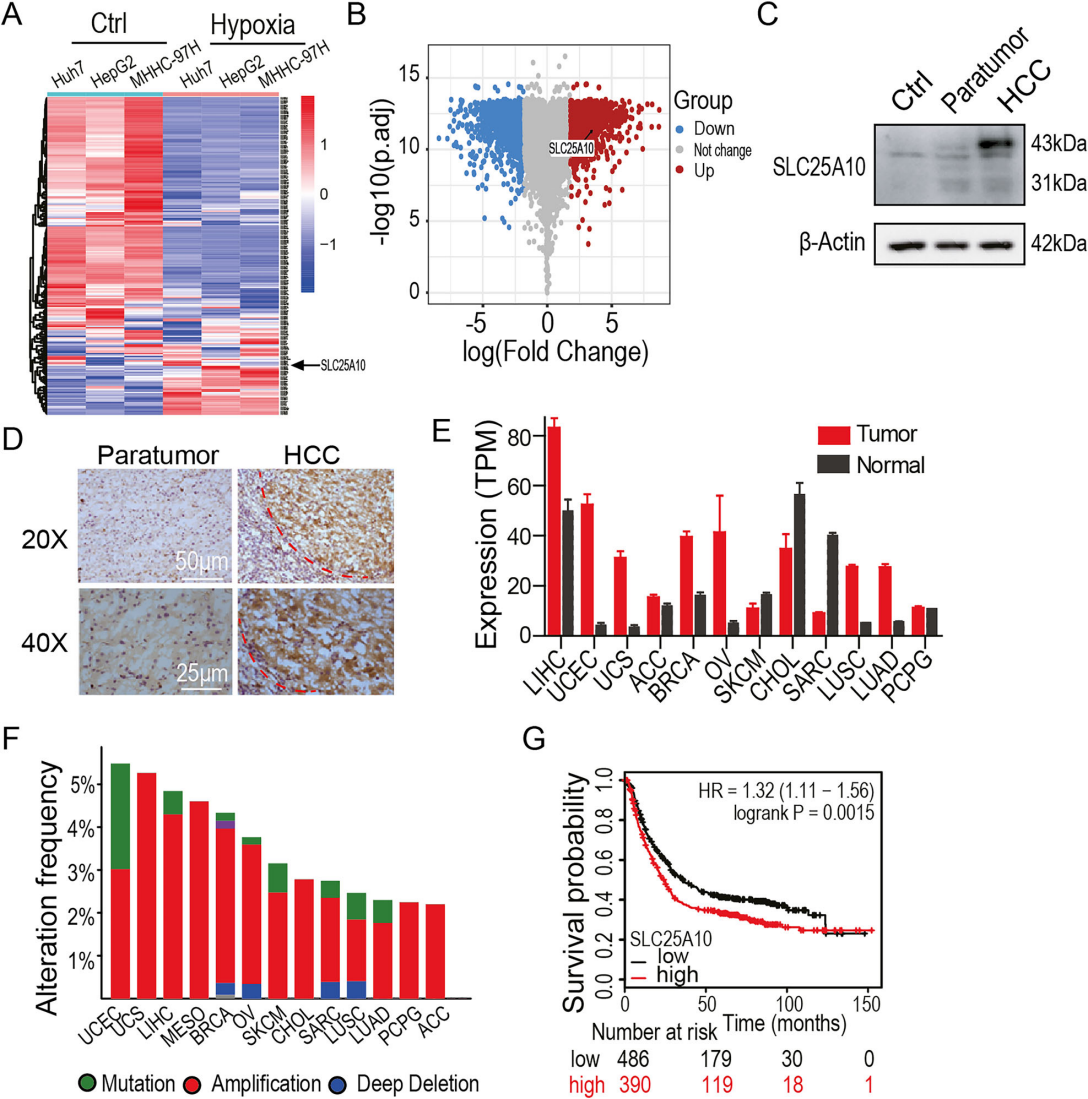
SLC25A10 plays a crucial role in HCC progression. **A** Heatmap displaying expression profiles of three HCC cell lines (Huh7, HepG2, and MHCC-97H) under hypoxic and normoxic conditions. RNA sequencing was performed in triplicate for each cell line. **B** Volcano plot showing differentially expressed genes in hypoxic V.S. normoxic Huh7 cells. **C** Immunoblotting analysis of SLC25A10 expression in representative clinical samples. **D** IHC staining of SLC25A10 in HCC tissues. **E** TCGA database analysis of SLC25A10 expression in multiple tumor types compared with matched normal tissues. Data presented as mean ± standard deviation; **p < 0.01, *p < 0.05, (two-tailed *t* test). **F** Frequency of *SLC25A10* genetic alterations in TCGA HCC cases. **G** Kaplan–Meier survival analysis of 476 HCC patients grouped by SLC25A10 expression. Notes: the SLC25A10 antibody was purchased from Sigma (Cat # HPA023048, Sigma-Aldrich, Darmstadt, Germany). **C**, **D** Show triplicate data. All statistical tests were two-sided tests.

First, we analyzed the gene expression profiles of HCC cells under hypoxic conditions. As shown in Fig. 1A, a heatmap illustrates the differential expression patterns among the three HCC cell lines Huh7, HepG2, and MHCC97H. A volcano plot (Fig. 1B) revealed that the mitochondrial carrier gene *SLC25A10* was significantly up-regulated in response to hypoxia. This upregulation was further validated by western blot analysis (Fig. 1C) and immunohistochemical (IHC) staining (Fig. 1D), both of which demonstrated markedly elevated SLC25A10 expression in clinical HCC tissues compared with adjacent non-tumorous tissues. Additionally, we evaluated SLC25A10 expression in several HCC cell lines available in our laboratory (Fig. S1A).

In addition, data from The Cancer Genome Atlas (TCGA; http://cancergenome.nih.gov/) revealed that *SLC25A10* is frequently amplified or mutated, and its mRNA expression is elevated across multiple tumor types, including HCC (Fig. 1E, F). Survival analysis further demonstrated that high SLC25A10 expression was significantly associated with poor prognosis in patients with HCC (Fig. 1G). Taken together, these findings suggested that SLC25A10 plays a critical role in HCC progression.

### Hypoxia-induced nuclear translocation of SLC25A10 isoform 3 is mediated by IPO7

**Fig. 2:**
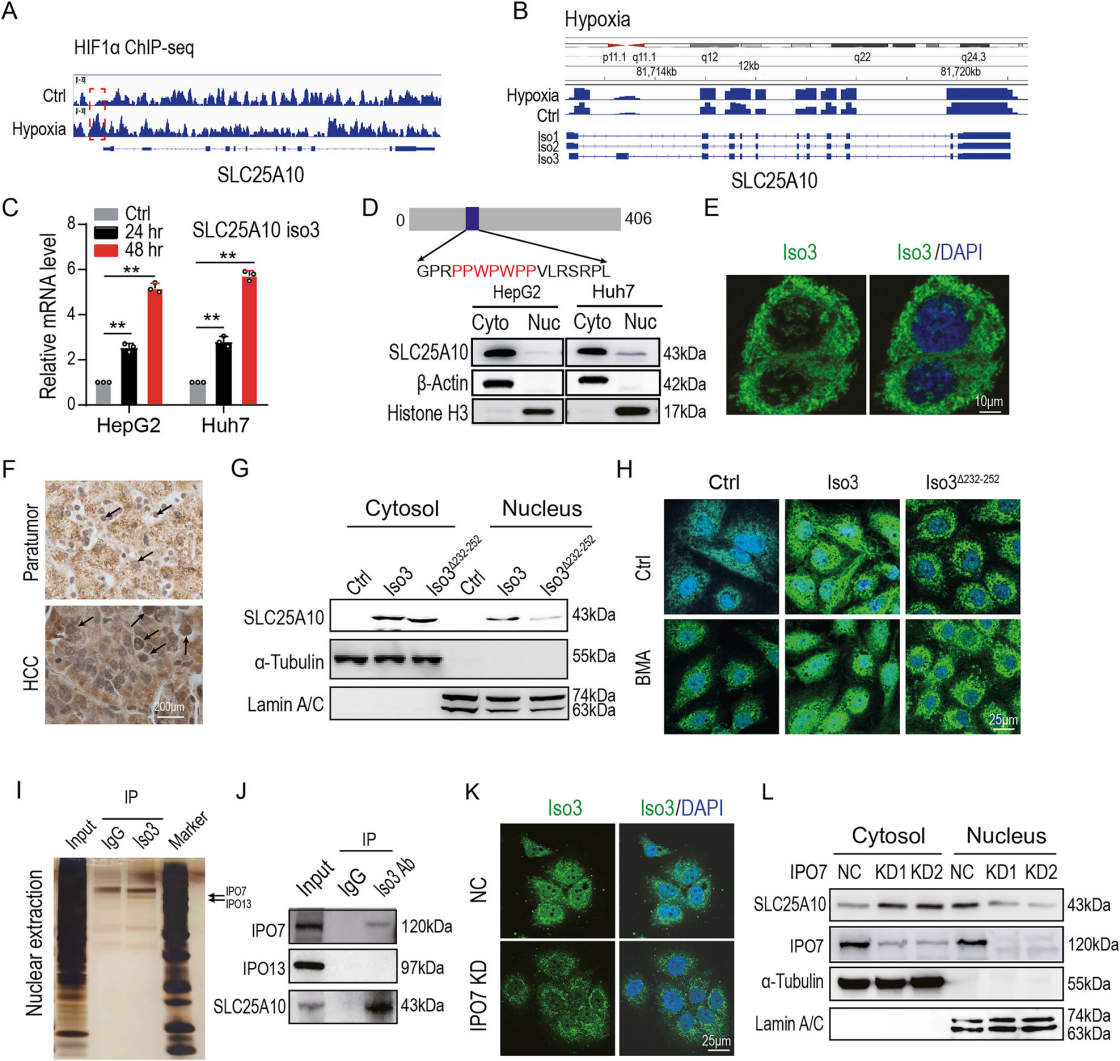
Hypoxia-induced nuclear translocation of SLC25A10 isoform 3 is mediated by IPO7. **A** ChIP-sequencing analysis of HIF1α binding at the *SLC25A10* promoter in Huh7 cells under hypoxic conditions (5% O_2_). **B** Alternative splicing analysis of *SLC25A10* transcripts in Huh7 cells under hypoxia. **C** *SLC25A10* isoform 3 expression detections in HepG2 and Huh7 cells under hypoxia by qPCR. Data are represented as mean ± SD (*n* = 3); **p < 0.01 (two-tailed *t* test). **D** Subcellular fractionation analysis of SLC25A10 localization under hypoxic conditions. **E** Confocal immunofluorescence microscopy of SLC25A10 isoform 3 in hypoxic Huh7 cells (×400 magnification). Arrows indicate nuclear localization. **F** IHC staining of SLC25A10 isoform 3 in HCC clinical samples (×100 magnification). Arrows denote nuclear staining. **G** Subcellular fractionation analysis of mutant SLC25A10 (ΔPPWPWPP) in Huh7 cells. **H** Confocal microscopy of truncated SLC25A10 isoform 3 in hypoxic Huh7 cells (×400 magnification). **I** Silver-stained SDS-PAGE gel of proteins co-immunoprecipitated with SLC25A10 isoform 3 from hypoxic HepG2 cell lysates. Candidate interactors were identified by mass spectrometry. **J** Validation of candidate binding partners co-immunoprecipitated with SLC25A10 isoform 3 under hypoxia. **K** Confocal microscopy of SLC25A10 isoform 3 in IPO7-depleted Huh7 cells under hypoxia (×400 magnification). **L** Subcellular fractionation analysis of SLC25A10 isoform 3 in IPO7-depleted HepG2 cells under hypoxia. Notes: Co-IP assay was used Pierce Immuno-Precipitation Kit (Cat # 26149, Thermo Scientific, NY, USA). The SLC25A10 antibody, specifically targeting the C-terminal extension region (amino acids 190–406), which is unique to isoform 3 was custom-produced by Abclonal (Cat # WG-03144D, Hubei, China) and was used for western blotting and IF. All experiments (**C**–**L**) were performed with three independent biological replicates. Scale bars are shown in relevant microscopy panels.

To investigate the effect of hypoxia on SLC25A10 expression, we first conducted chromatin immunoprecipitation sequencing (ChIP-seq) using an HIF1α-specific antibody. As shown in Fig. 2A, hypoxic conditions enhanced HIF1α binding to the *SLC25A10* promoter region. Transcriptomic profiling of the HCC cell lines HepG2 and Huh7 further revealed that hypoxia induced a splicing shift from *SLC25A10* isoform 2 to isoform 3 (Fig. 2B and S2A), which was subsequently validated by quantitative PCR (qPCR) (Fig. 2C).

Protein sequence analysis indicated that SLC25A10 isoform 3 contains a putative nuclear localization signal (NLS), PPWPWPP, suggesting its potential for nuclear translocation. Western blot analysis of the nuclear fractions confirmed the presence of SLC25A10 isoform 3 in the nucleus (Fig. 2D). This finding was further supported by confocal immunofluorescence imaging, which demonstrated nuclear localization of SLC25A10 isoform 3 (Fig. 2E). Immunohistochemical analysis of HCC tissues also revealed increased nuclear accumulation of SLC25A10 isoform 3 compared with that in adjacent non-tumorous tissues (Fig. 2F).

To evaluate the role of the PPWPWPP motif in nuclear localization, we analyzed a truncated version of SLC25A10 isoform 3 that lacked this sequence. Overexpression of full-length isoform 3 resulted in prominent nuclear accumulation, whereas the truncated variant of SLC25A10 isoform 3 failed to localize to the nucleus (Fig. 2G), as confirmed by immunofluorescence microscopy. Notably, treatment with butylmalonate (BMA), a mitochondrial carrier inhibitor that inhibits SLC25A10-mediated transport [8], did not affect the nuclear translocation of SLC25A10 (Fig. 2H). This suggests that nuclear translocation of SLC25A10 occurs independently of its canonical transport function.

To identify the nuclear import machinery responsible for SLC25A10 isoform 3 translocation, we performed co-immunoprecipitation followed by mass spectrometry (Co-IP/MS) to screen for the associated nuclear transporters. Among these candidate proteins, IPO7 and IPO13 were identified as potential interactors [10, 11] (Fig. 2I). However, subsequent western blot analysis confirmed a specific interaction between IPO7, but not IPO13, and SLC25A10 isoform 3 (Fig. 2J). Confocal microscopy further revealed that, under hypoxia, endogenous IPO7 and SLC25A10 isoform 3 co-localize in a discrete perinuclear region (Fig. S2B). Notably, knockdown of IPO7 significantly reduced the nuclear accumulation of SLC25A10 isoform 3, as demonstrated by both western blotting and immunofluorescence staining (Fig. 2K, L), indicating that IPO7 is essential for its nuclear localization.

### The SLC25A10 isoform 3 attenuates etoposide-induced HCC apoptosis

**Fig. 3:**
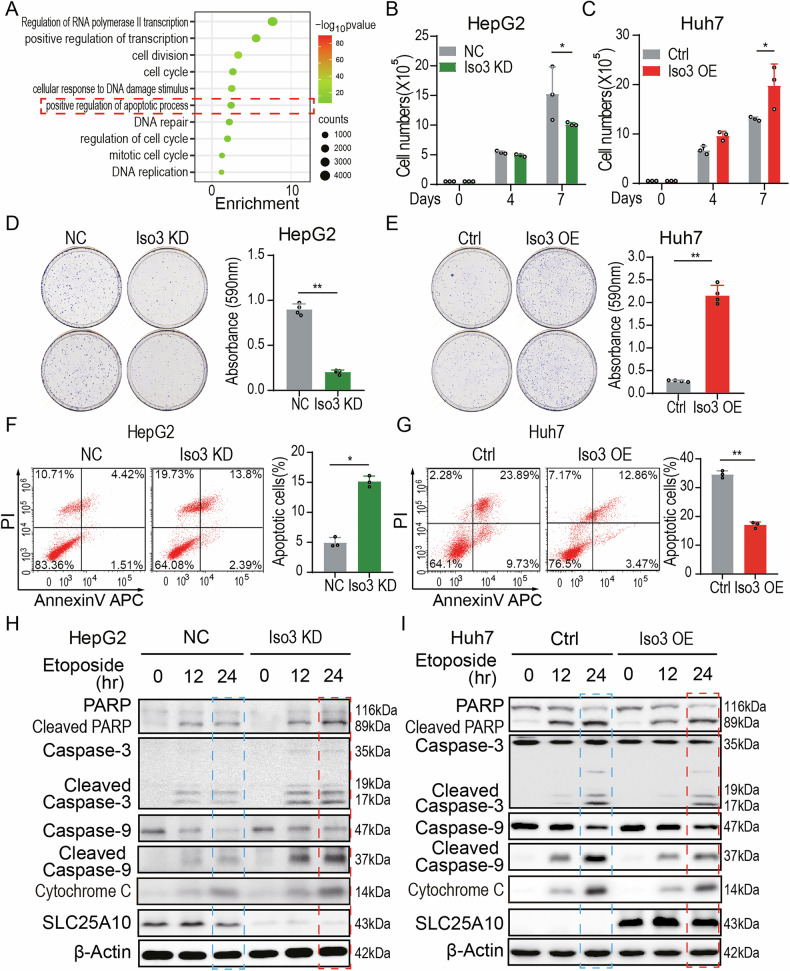
The SLC25A10 isoform 3 attenuates Etoposide-induced HCC apoptosis. **A** Gene ontology (GO) analysis of differentially expressed genes in Huh7 cells under hypoxia. **B** Growth curve of HepG2 cells following knockdown of *SLC25A10* isoform 3. The 10^5^ cells were seeded at baseline and treated with Etoposide (2.5 μM). **C** Growth curve of Huh7 cells overexpressing SLC25A10 isoform 3. The 10^5^ cells were seeded at baseline and treated with Etoposide (2.5 μM). **D** Colony formation assay in HepG2 cells depleted of SLC25A10 isoform 3. The 3 × 10^3^ cells were seeded and treated with Etoposide (2.5 μM) for 10 days. **E** Colony formation assay in Huh7 cells overexpressing SLC25A10 isoform 3. The 2 × 10^3^ cells were seeded and treated with Etoposide (2.5 μM) for 10 days. **F** Flow cytometry analysis of apoptosis in HepG2 cells following *SLC25A10* isoform 3 knockdown. Cells were treated with Etoposide (15 μM) for 24 hours. **G** Flow cytometry analysis of apoptosis in Huh7 cells overexpressing SLC25A10 isoform 3. Cells were treated with Etoposide (15 μM) for 24 h. **H** Apoptotic signaling was assessed by western blotting in HepG2 cells depleted of SLC25A10 isoform 3. Cells were treated with Etoposide (15 μM) for 0, 12, or 24 h. **I** Apoptotic signaling was assessed by western blotting in Huh7 cells overexpressing SLC25A10 isoform 3. Cells were treated with Etoposide (15 μM) for 0, 12, or 24 h. Notes: The SLC25A10-Iso3 antibody (Cat # WG-03144D, Abclonal, Hubei, China) was used for western blotting. Data are represented as mean ± SD; **p < 0.01, *p < 0.05. All experiments (**B**–**I**) were performed in triplicate.

To evaluate the role of SLC25A10 isoform 3 in HCC progression, we performed Gene Ontology (GO) analysis of differentially expressed genes in HCC cells exposed to hypoxic conditions. As shown in Fig. 3A, apoptotic signaling pathways were significantly activated in hypoxic Huh7 cells.

**Fig. 4:**
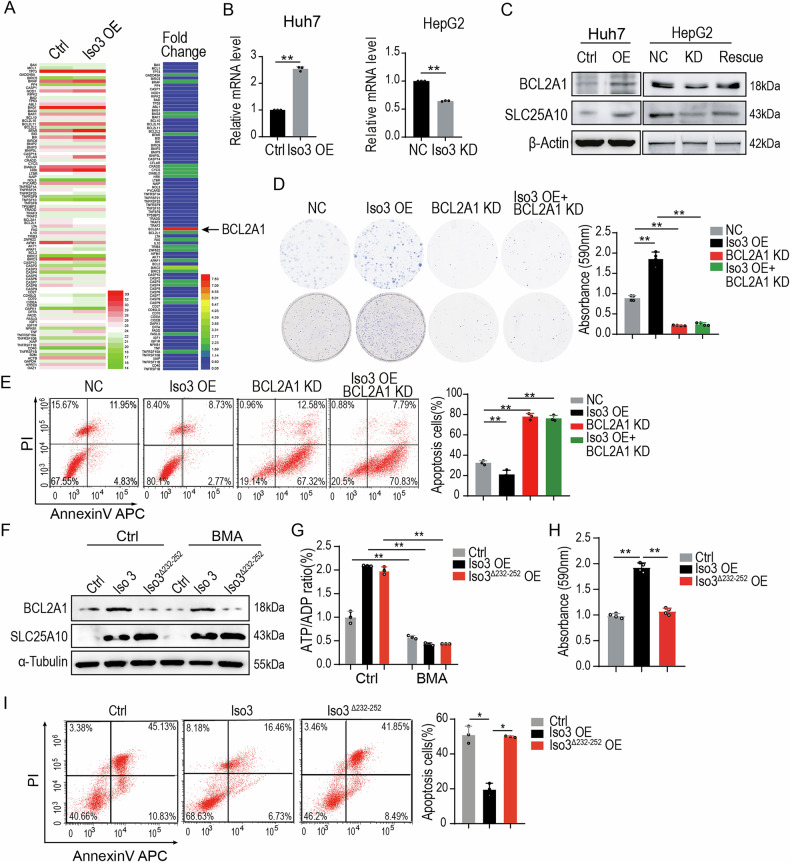
SLC25A10 isoform 3 upregulates BCL2A1 expression. **A** Apoptosis-related protein expression profiles in Huh7 cells modulated by SLC25A10 isoform 3. **B** qPCR validation of BCL2A1 expression in Huh7 and HepG2 cells overexpressing or depleted of SLC25A10 isoform 3. **C** Immunoblot analysis confirming BCL2A1 protein levels in Huh7 and HepG2 cells overexpressing or depleted of SLC25A10 isoform 3. **D** Colony formation assay assessing the impact of *BCL2A1* knockdown on Huh7 cells overexpressing SLC25A10 isoform 3. The 10^3^ cells were treated with Etoposide (2.5 μM) for 10 days. **E** Flow cytometry analysis of apoptosis in BCL2A1-depleted Huh7 cells. Cells were treated with Etoposide (15 μM) for 24 h. **F** Immunoblot analysis of BCL2A1 expression in Huh7 cells treated with BMA (8 mM, 24 h), a selective inhibitor of SLC25A10 transport activity. **G** ATP/ADP ratio measurement in Huh7 cells following BMA treatment (8 mM) for 24 h. **H** Colony formation assay evaluating Etoposide sensitivity in Huh7 cells. Cells were treated with Etoposide (2.5 μM) for 10 days. **I** Flow cytometry analysis of etoposide-induced apoptosis in Huh7 cells. Cells were treated with Etoposide (15 μM) for 24 h. Note: all experiments were performed in triplicate; Data are represented as mean ± SD. **p < 0.01, *p < 0.05.

To investigate the role of SLC25A10 isoform 3 in apoptosis, we assessed its effects on HCC cells that either depleted or overexpressed this isoform. As shown in Fig. 3B and S3A, knockdown of *SLC25A10* isoform 3 significantly decreased the viability of HepG2 cells treated with etoposide, whereas its overexpression enhanced Huh7 cell proliferation (Fig. 3C and S3B). Colony formation assays further demonstrated that silencing *SLC25A10* isoform 3 reduced the number of HepG2 colonies following etoposide treatment (Fig. 3D), whereas overexpression of isoform 3, but neither isoform 2 nor isoform 1, increased colony formation in Huh7 cells (Fig. 3E and S3C). Flow cytometric analysis revealed that depletion of BCL2A1, a known anti-apoptotic protein, markedly increased etoposide-induced apoptosis of HCC cells. In contrast, overexpression of SLC25A10 isoform 3 did not significantly attenuate apoptosis under these conditions (Fig. 4E).

To elucidate the mechanism by which SLC25A10 isoform 3 regulates HCC cell survival, we quantified apoptosis using flow cytometry in Etoposide-treated cells. In HepG2 cells, knockdown of isoform 3 increased the apoptosis rate from 5.93% to 16.19% (Fig. 3F), whereas in Huh7 cells, overexpression of isoform 3, but not isoform 1 or 2, reduced apoptosis from 33.62% to 16.33% (Fig. 3G and S3D).

Next, we examined the activation of the apoptotic pathways in HCC cells. Parental Huh7 cells exhibited elevated levels of cleaved PARP, cleaved caspase-3, and cleaved caspase-9 following etoposide treatment compared to HepG2 cells, which expressed higher levels of SLC25A10 (Fig. 3H, I, left vs. right blue frames). Consistent with these observations, SLC25A10 isoform 3 knockdown in HepG2 cells enhanced cytochrome C release and increased the activation of PARP, caspase-3, and caspase-9 (left blue frame vs. left red frame in Fig. 3H). Conversely, overexpression of SLC25A10 isoform 3 in Huh7 cells reduced the levels of these apoptotic markers relative to those in parental cells (right blue frame vs. right red frame in Fig. 3I). Collectively, these results suggest that SLC25A10 isoform 3 promotes resistance to etoposide-induced apoptosis in HCC cells and may contribute to tumor cell survival under hypoxic stress.

### SLC25A10 isoform 3 upregulates BCL2A1 expression

To investigate the mechanism by which SLC25A10 isoform 3 modulates etoposide-induced apoptosis, we analyzed the transcriptional profile of apoptosis-related genes in Huh7 cells overexpressing this isoform (Fig. 4A). Among the differentially expressed genes, *BCL2A1* was identified as the most significantly upregulated gene. This upregulation was subsequently validated by qPCR and Western blot analysis (Fig. 4B, C).

Functionally, colony formation assays demonstrated that *BCL2A1* knockdown increased the sensitivity of Huh7 cells to etoposide, even in the presence of SLC25A10 isoform 3 overexpression (Fig. 4D). Similarly, flow cytometry analysis revealed that BCL2A1 depletion enhanced etoposide-induced apoptosis, whereas overexpression of the SLC25A10 isoform 3 did not significantly attenuate this effect (Fig. 4E).

To determine whether the anti-apoptotic effect of SLC25A10 isoform 3 depends on its mitochondrial transporter function, the cells were treated with BMA. As shown in Fig. 4F, G, although BMA impaired mitochondrial function in Huh7 cells, it did not affect SLC25A10 isoform 3-induced BCL2A1 expression, irrespective of whether the cells overexpressed wild-type isoform 3 or a truncated mutant lacking the PAWAWAP motif.

Consistent with these findings, both colony formation and flow cytometry analyses demonstrated that deletion of the PAWAWAP motif did not compromise the protective effect of SLC25A10 isoform 3 against Etoposide-induced apoptosis (Fig. 4H, S4A and 4I). Collectively, these results indicate that the anti-apoptotic function of SLC25A10 isoform 3 is independent of its canonical mitochondrial transporter activity. Instead, isoform 3 exerts a distinct and critical regulatory role, likely mediated by the upregulation of BCL2A1, in modulating apoptosis in HCC cells.

### SLC25A10 isoform 3 interacts with the transcription factor CEBPB to regulate BCL2A1 expression

**Fig. 5:**
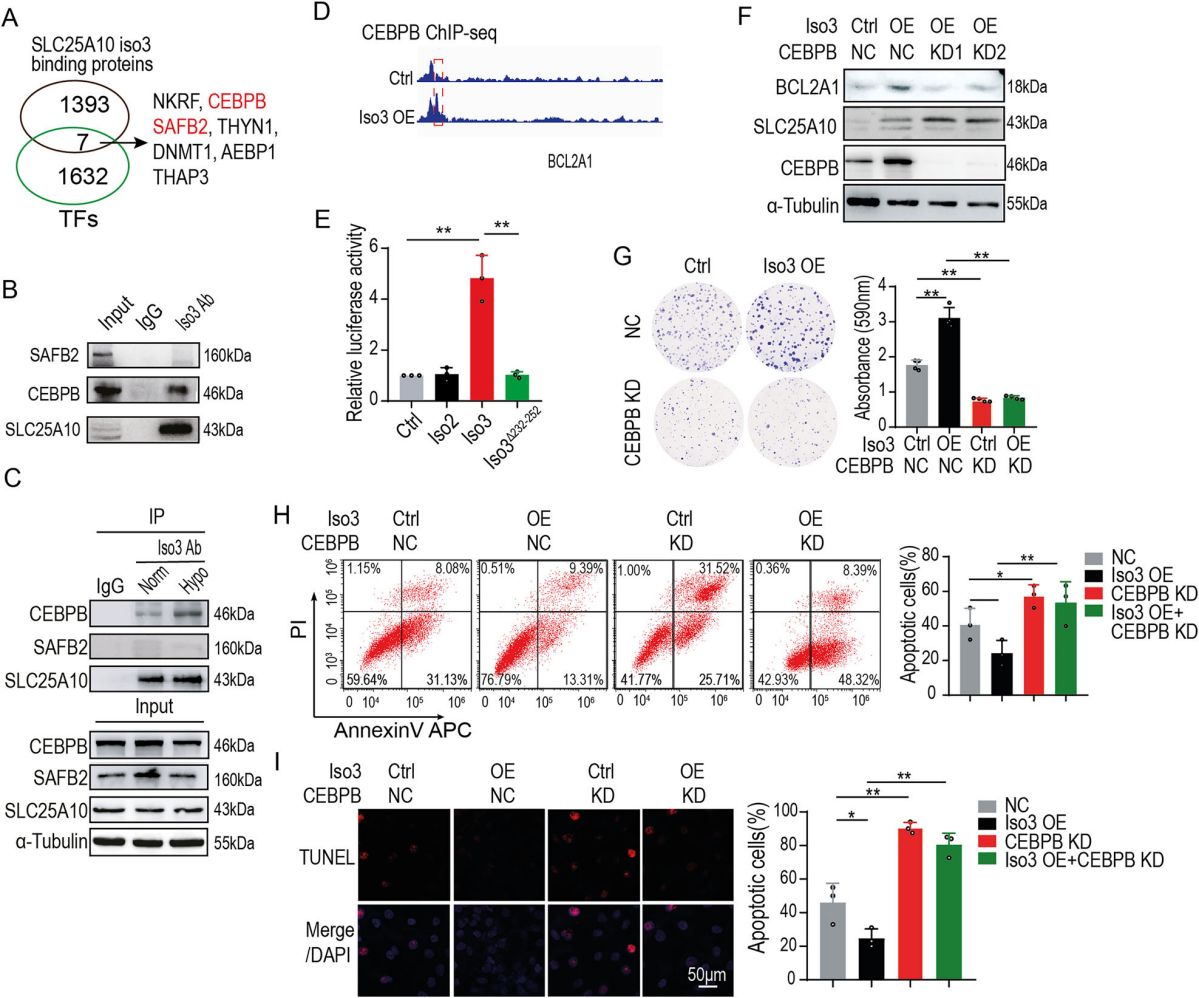
SLC25A10 isoform 3 interacts with the transcription factor CEBPB to regulate BCL2A1 expression. **A** Overlap analysis between SLC25A10 isoform 3-binding proteins (defined by protein mass spectrometry) and known transcription factors. **B** Immunoblotting analysis of transcription factors co-immunoprecipitated with SLC25A10 isoform 3 in hypoxic HepG2 cells. **C** Immunoblot analysis of SLC25A10 isoform 3-interacting transcription factors in HepG2 cells under normoxia or hypoxia. **D** ChIP-seq analysis of CEBPB binding at the locus of *BCL2A1* in Huh7 cells. **E** The impact of SLC25A10 isoform 3 on *BCL2A1* promoter activity was assessed using a dual-luciferase reporter assay. Reporter constructs were generated by subcloning a -641 to -19 bp fragment of the *BCL2A1* gene promoter into the pGL4.10 luciferase reporter vector. The position of the SLC25A10 isoform 3-binding motif is indicated. **F** Immunoblot analysis of BCL2A1 expression in Huh7 cells overexpressing SLC25A10 isoform 3 with or without the CEBPB depletion. **G** Colony formation assay assessing the effect of CEBPB depletion on SLC25A10 isoform 3-mediated apoptosis resistance. The 10^3^ cells were treated with Etoposide (2.5 μM) for 10 days. **H** Flow cytometry analysis of apoptosis in CEBPB-depleted Huh7 cells overexpressing SLC25A10 isoform 3. Cells were treated with Etoposide (15 μM) for 24 h. **I** TUNEL assay quantifying apoptosis in CEBPB-depleted Huh7 cells overexpressing SLC25A10 isoform 3. Cells were treated with Etoposide (15 μM) for 24 h. Note: All experiments were performed in triplicate; Data are represented as mean ± SD. **: *p* < 0.01, *: *p* < 0.05.

To identify the transcription factor(s) responsible for BCL2A1 upregulation by SLC25A10 isoform 3, we performed protein mass spectrometry following the co-immunoprecipitation of the nuclear fraction of cell lysates. As shown in Fig. 5A, SLC25A10 isoform 3 was found to be associated with seven transcription-related proteins, including transcription factors CEBPB and SAFB2. However, Western blot analysis of the co-immunoprecipitated complexes confirmed that only CEBPB physically interacted with SLC25A10 isoform 3 (Fig. 5B), and this interaction was enhanced under hypoxic conditions (Fig. 5C).

ChIP-seq further revealed enrichment of CEBPB in the promoter region of *BCL2A1* in Huh7 cells overexpressing SLC25A10 isoform 3 (Fig. 5D). Consistent with this, a luciferase reporter assay targeting the *BCL2A1* promoter region (-641 to -19 bp) demonstrated that SLC25A10 isoform 3, but not isoform 2 or mutant isoform 3, enhanced CEBPB binding to the *BCL2A1* promoter (Fig. 5E), supporting a direct transcriptional regulatory relationship. To assess whether the anti-apoptotic effect of SLC25A10 isoform 3 depended on CEBPB, we evaluated BCL2A1 expression in CEBPB-depleted Huh7 cells. Western blot analysis showed that knockdown of CEBPB significantly attenuated the isoform 3-induced upregulation of BCL2A1 (Fig. 5F).

Furthermore, depletion of CEBPB abolished the protective effect of SLC25A10 isoform 3 against etoposide-induced apoptosis, as demonstrated by colony formation, flow cytometry, and TUNEL assays (Fig. 5G–I). These findings indicate that SLC25A10 isoform 3 regulates BCL2A1 expression via its interaction with the transcription factor CEBPB, thereby contributing to apoptosis resistance in HCC cells.

### Disrupting the interaction between SLC25A10 isoforms and IPO7 enhances the HCC sensitivity to etoposide in mouse models

**Fig. 6:**
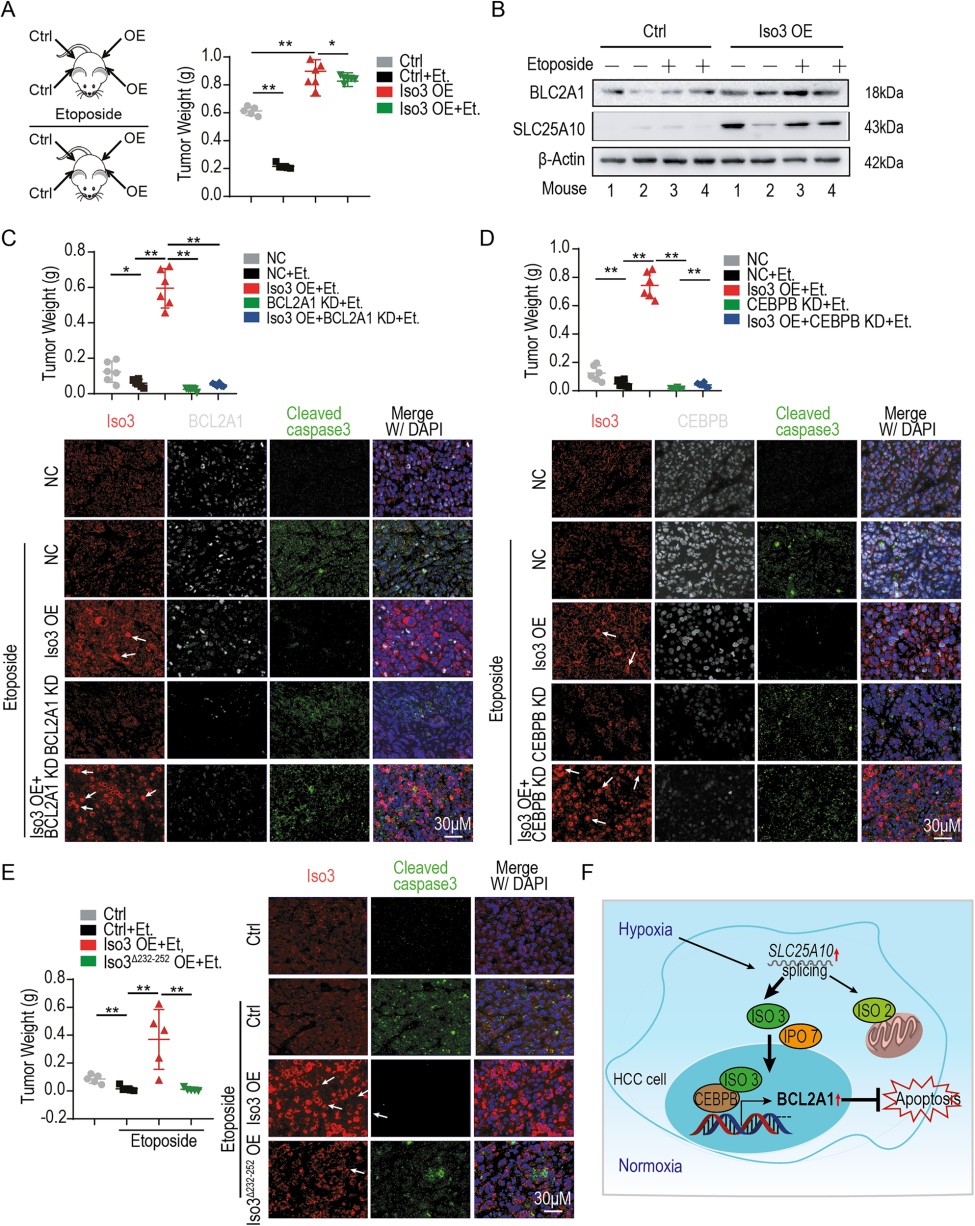
Disrupting the interaction between SLC25A10 isoforms and IPO7 enhances the HCC sensitivity to etoposide in mouse models. **A** Tumor weights in PCX mice xenografted with Huh7 cells overexpressing SLC25A10 isoform 3 (5 × 10^6^ cells/injection), with or without etoposide treatment (20 mg/kg, 21 days). **B** Immunoblotting analysis of BCL2A1 in representative xenografts with or without the SLC25A10 isoform 3 overexpression. **C** Tumor growth in PCX mice xenografted with BCL2A1-depleted Huh7 cells treated with etoposide (arrows indicate SLC25A10 isoform 3 localization). **D** Tumor growth in PCX mice xenografted with CEBPB-depleted Huh7 cells treated with etoposide (arrows indicate SLC25A10 isoform 3 localization). **E** Tumor growth in PCX mice xenografted with Huh7 cells expressing mutant SLC25A10 isoform 3 exposed to etoposide (arrows indicate SLC25A10 isoform 3 localization). **F** The working model of SLC25A10 isoform 3. Note: The SLC25A10-Iso3 antibody (Cat # WG-03144D, Abclonal, Hubei, China) was used for western blotting and mIHC. All experiments were performed in triplicate. Data are presented as mean ± SD. **p < 0.01, *p < 0.05.

To evaluate the role of SLC25A10 isoform 3 in modulating the chemotherapeutic efficacy of etoposide in HCC, we utilized a xenograft mouse model. As shown in Fig. 6A and S5A, Huh7 cells with or without SLC25A10 isoform 3 overexpression were subcutaneously injected into the axillary and inguinal regions of the mice. Tumors derived from control Huh7 cells were sensitive to etoposide treatment; however, overexpression of SLC25A10 isoform 3 significantly diminished the therapeutic efficacy of the drug. Consistently, BCL2A1 protein levels were elevated in tumors overexpressing the SLC25A10 isoform 3 (Fig. 6B).

To further elucidate the role of BCL2A1 in this regulatory pathway, we analyzed xenografts derived from Huh7 cells with *BCL2A1* knockdown with or without SLC25A10 isoform 3 overexpression. As anticipated, BCL2A1 depletion enhanced tumor sensitivity to etoposide, even in the context of SLC25A10 isoform 3 overexpression (Fig. 6C and S5B). Multiplex immunohistochemistry analysis corroborated these findings. Although SLC25A10 isoform 3 upregulated BCL2A1 expression and suppressed cleaved caspase-3 levels, it failed to counteract the increased caspase-3 activation resulting from *BCL2A1* knockdown (Fig. 6C).

We evaluated the role of CEBPB in this regulatory axis. Huh7 cells, with or without SLC25A10 isoform 3 overexpression and concurrent *CEBPB* knockdown, were implanted into mice. Consistent with prior observations, overexpression of SLC25A10 isoform 3 reduced sensitivity to etoposide. However, CEBPB depletion restored etoposide-induced apoptosis, effectively abolishing the protective effect mediated by SLC25A10 isoform 3 (Fig. 6D and S5C). Multiplex immunohistochemical analysis further confirmed that SLC25A10 isoform 3 was unable to suppress cleaved caspase-3 levels in the absence of CEBPB, supporting a CEBPB-dependent mechanism of anti-apoptotic regulation.

Finally, we investigated whether disrupting the nuclear import of SLC25A10 isoform 3 via IPO7 enhanced etoposide sensitivity. To this end, we generated a mutant isoform 3 lacking the IPO7-binding motif (Δ232-252; PPWPWPP) and compared its effects with those of the wild-type isoform in a xenograft model. As shown in Fig. 6E and S5D, tumors expressing the binding-deficient mutant exhibited increased sensitivity to etoposide compared to those expressing the wild-type isoform 3. Multiplex immunohistochemistry analysis further revealed that tumors harboring the mutant isoform failed to significantly suppress cleaved caspase-3 levels, indicating a loss of anti-apoptotic function.

Collectively, these findings suggest that the hypoxia-induced splicing of SLC25A10 into isoform 3 facilitates IPO7-mediated nuclear translocation, where it regulates BCL2A1 expression. Thus, targeting the SLC25A10-IPO7 interaction may represent a promising therapeutic strategy to enhance the efficacy of chemotherapy in HCC.

## Discussion

Previous studies have established that the mitochondrial carrier SLC25A10 is predominantly localized in the mitochondria, where it plays a critical role in supplying malate for citrate synthesis. SLC25A10 has also been implicated in the enhancement of cancer cell resistance to radiation-induced damage [8]. However, the precise molecular mechanisms underlying this protective effect remain unclear.

In this study, we identified an alternative splicing event that generates isoform 3 of SLC25A10 in HCC. This event is markedly upregulated under hypoxic conditions, a hallmark of the tumor microenvironment. Hypoxia stabilize the hypoxia-induced factors, such as HIF1α [12], which regulates widespread transcriptional changes [13–16] and, as emerging evidence shows, modulates alternative mRNA splicing [17–20]. Our findings suggest that hypoxia induces SLC25A10 splicing through an HIF1α-dependent mechanism, thereby enhancing chemoresistance.

In contrast to canonical mitochondrial localization, isoform 3 translocates to the nucleus through interaction with the nuclear import receptor IPO7. Within the nucleus, SLC25A10 isoform 3 associates with the transcription factor CEBPB, thereby promoting transcription of the anti-apoptotic gene *BCL2A1* (Fig. 6F). This nuclear function of isoform 3 enhances HCC cell resistance to the chemotherapeutic agent, etoposide. Notably, disruption of the isoform 3-IPO7 interaction significantly sensitized HCC tumors to etoposide in vivo, suggesting a promising therapeutic strategy via disrupting nuclear translocation or targeting the transcriptional complex to improve the efficacy of chemotherapy in HCC.

Regarding the nuclear translocation of SLC25A10 isoform 3, it remains unclear whether specific post-translational modifications (PTMs) are necessary for its interaction with IPO7 and its subsequent nuclear import. Future investigations should focus on determining whether hypoxia regulates such PTMs and, if so, identify the enzymes and signaling pathways involved. Additionally, our data indicate that isoform 3 enhances the chromatin-binding affinity of CEBPB; however, the precise domain or motif within SLC25A10 isoform 3 that mediates this interaction has yet to be elucidated. Unraveling the molecular interface between isoform 3 and CEBPB is essential for the rational development of small-molecule inhibitors aimed at disrupting this interaction, thereby potentially restoring chemosensitivity in resistant HCC.

The discovery of a hypoxia-induced, nuclear-localized SLC25A10 isoform 3 establishes it as both a companion diagnostic biomarker and a therapeutic target in HCC. Its splice index (isoform 3/total *SLC25A10*), which can be quantified within hours via RNAscope or digital PCR on routine biopsy samples, identifies tumors characterized by hypoxia-driven chemoresistance and directly guides therapeutic adjustments—including switching to non-platinum regimens or enrolling patients in targeted clinical trials [21, 22]. Further supporting this paradigm, recent multi-cancer studies demonstrate that elevated total SLC25A10 expression also suppresses cellular senescence and ferroptosis, while enhancing cisplatin resistance in HCC, prostate, and cervical cancers. These findings imply that the mitochondrial SLC25A10 carrier and its nuclear isoform 3 function in tandem: the former blunts iron-dependent cell death, whereas the latter hijacks the CEBPB–BCL2A1 transcriptional axis to block apoptotic pathways [23–25]. Consequently, a composite scoring system integrating total SLC25A10 protein levels (measurable by IHC or proteomic profiling) with the isoform 3 splice index enables stratification of patients into low-, intermediate-, and high-risk categories for platinum-based treatment failure. This scoring framework provides a quantitative basis to select upfront therapeutic strategies (platinum monotherapy, non-platinum regimens, or ferroptosis-inducing combination therapies) and to identify patient subsets most likely to benefit from isoform 3-specific or pan-*SLC25A10* inhibitors. Concomitantly, targeting protein–protein interaction interfaces has emerged as a promising therapeutic strategy for diverse diseases [26–29]. The isoform 3-IPO7-CEBPB-BCL2A1 signaling axis harbors two readily druggable nodes: (1) blockade of nuclear import via cell-penetrant IPO7-competing peptides or small-molecule inhibitors, and (2) disruption of CEBPB co-activation using structure-guided antagonists. Both approaches restore etoposide sensitivity without inducing additional off-target toxicity and can be seamlessly combined with standard chemotherapy, ferroptosis inducers, or immunotherapies—thus yielding a companion diagnostic-linked therapeutic platform primed for clinical translation.

In conclusion, this study revealed a previously unrecognized nuclear function of the mitochondrial dicarboxylate carrier SLC25A10 in transcriptional regulation under hypoxic conditions, distinct from its canonical mitochondrial role. These findings broaden our understanding of SLC25A10 biology and reveal a novel mechanism that drives hypoxia-induced chemoresistance in HCC. Targeting this axis-through inhibition of nuclear translocation or transcriptional co-activation-holds significant promise for improving therapeutic outcomes of chemoresistant HCC patients.

## Material and method

### Cell lines and reagents

All the cell lines were maintained at 37 °C in a humidified atmosphere containing 5% CO₂. For hypoxic conditions, cells were cultured in 1% O₂ with 5% CO₂. Human HCC cell lines HepG2 and Huh7, as well as HEK-293T cells were obtained from the American Tissue culture collection (ATCC, VA, USA). The cells were grown in Dulbecco’s Modified Eagle’s medium (DMEM; Cat# L110KJ, BasalMedia, Shanghai, China) supplemented with 10% fetal bovine serum (Cat # 10270-106, Gibco, NY, USA) and 50 IU/mL penicillin-streptomycin (Cat # S110JV, BasalMedia, Shanghai, China). All cells were authenticated by STR profiling and tested for mycoplasma contamination.

Commercial antibodies used in this study included: BCL2A1 (Cat # 14093, CST, MA, USA), Caspase-3 (Cat # 14420, CST, MA, USA), Cleaved Caspase-3 (Cat # 9664, CST, MA, USA), Caspase-9 (Cat # 9508, CST, MA, USA), Cleaved Caspase-9 (Cat # 52873, CST, MA, USA), CEBPB (Cat # PA5-27244, Invitrogen, CA, USA), Cytochrome c (Cat # 4272, CST, MA, USA), IPO7 (Cat # 28289-1-AP, Proteintech, Hubei, China), RPAP (Cat # 9542, CST, MA, USA), Cleaved RPAP (Cat # 5625, CST, MA, USA), SAFB2 (Cat # A4330, Abclonal, Hubei, China), SLC25A10 (Cat # HPA023048, Sigma-Aldrich, Darmstadt, Germany), SLC25A10 (Cat # WG-03144D, Abclonal, Hubei, China) for specifically targeting the C-terminal extension region (amino acids 190 - 406) of SLC25A10- Iso3, Pol II (Cat # sc-899, Santa Cruz, TX, USA), Lamin A/C (Cat # 2032T, CST, MA, USA), Histone H3 (Cat # AM8433, Abcepta, CA, USA), β-actin (Cat # sc-47778, Santa Cruz, TX, USA), α-Tubulin (Cat # 11224-1-AP, Proteintech, Hubei, China).

### Plasmid construction and transfections

The open reading frame (ORF) of SLC25A10 isoform 3 (NM_001270953.2) was cloned into the lentiviral vector pCDH-CMV-MCS-EF1-Puro to generate FLAG-tagged constructs. Site-directed mutagenesis was performed using AccuPrime Pfx DNA Polymerase (Cat # 12344024, Invitrogen, CA, USA) to create the SLC25A10-V3^Δ232-252^ mutant.

The following shRNA sequences were cloned into the pLKO.1-puro vector:

SLC25A10 isoform 3: TGCTAGCTCTGCACTTCGTGT

BCL2A1: GCCAGAACACTATTCAACCAA

IPO7: #1 GCTAACAAGAAGATGTCTGAT, #2 GCACTGACTCACGGTCTTAAT

CEBPB: #1 CCCGTGGTGTTATTTAAAGAA, #2 CCTGCCTTTAAATCCATGGAA

Scramble control: GAATTACTCCTAGAACCGC

Lentiviral particles were generated by co-transfecting HEK-293T cells with shRNA plasmids and packaging plasmids psPAX2 and pMD2.G. Forty-eight hours post-infection, stable cell lines were selected using puromycin (1 μg/mL) or blasticidin (10 μg/mL). Knockdown and overexpression efficiencies were confirmed using quantitative real-time PCR and immunoblotting, respectively.

### Western blot analysis

Cells at 80-90% confluence were harvested and washed with PBS. Nuclear and cytoplasmic fractions were isolated using the Nuclear and Cytoplasmic Protein Extraction Kit (Cat # PK10014, Proteintech, Hubei, China) following the manufacturer’s instructions. Total protein was extracted using RIPA lysis buffer supplemented with protease inhibitors (1 mM PMSF, 1 mg/L aprotinin, leupeptin, and pepstatin) and phosphatase inhibitors (1 mM Na₃VO₄ and 10 mM NaF). After centrifugation at 12,000 × *g* for 20 min at 4 °C, the protein concentrations were quantified using a BCA assay kit (Cat # MA0082-1, Meilunbio, Liaoning, China).

Protein samples (20-40 μg) were resolved using 10% SDS-PAGE and transferred onto PVDF membranes. The membranes were blocked and incubated with primary antibodies overnight at 4 °C, followed by incubation with the appropriate secondary antibodies. Protein bands were detected using a LAS 4000 imaging system (GE Healthcare).

### Real-time PCR

Total RNA was extracted using the TRIzol reagent. cDNA was synthesized from 1 μg of total RNA using the Evo M-MLV RT Reaction Mix (Cat # AG11728, AG, Hunan, China), according to the manufacturer’s protocol. Quantitative PCR was conducted with 2× SYBR Green Master Mix (Cat # AG11719, AG, Hunan, China) using the following primer pairs:

*SLC25A10* isoform 1: Forward: 5’-TTATTGCAGCCGCTGGTGAC-3’; Reverse: 5’-GTCTTCAGCACATCCAGGGG-3’

*SLC25A10* isoform 2: Forward: 5’-CTTTGTCGCCAGCTTTATTG-3’; Reverse: 5’-TCACCAGCGGCTGCAATAA-3’

*SLC25A10* isoform 3: Forward: 5’-GGGGAGTATCAGGGCGTTTT-3’; Reverse: 5’-CAAAAGTGAGCACGGTGTGG-3’

*BCL2A1*: Forward: 5’-GCGGGAAATCGTGCGTGACATT-3’; Reverse: 5’-GATGGAGTTGAAGGTAGTTTCG-3’

*GAPDH* (internal control): Forward: 5’-ACCCAGAAGACTGTGGATGG-3’; Reverse: 5’-CAGTGAGCTTCCCGTTCAG-3′

Amplification and detection were performed on an ABI 7500 Sequence Detection System (Applied Biosystems). Relative expression levels were calculated using the 2^(-ΔΔCt)^ method, with *GAPDH* as the reference gene.

### Cell proliferation analysis

For proliferation analysis, cells were seeded in 60-mm dishes at a density of 2 × 10⁵ cells per dish in DMEM supplemented with 10% FBS and maintained at 37 °C with 5% CO₂. The cell numbers were counted at the indicated time points using a Countess automated cell counter (Invitrogen, Grand Island, NY, USA).

For colony formation assays, cells were plated in 6-well plates at a density of 1 × 10³ cells/well and cultured for 10 days under standard conditions. The colonies were fixed with 4% paraformaldehyde for 30 min and stained with 2.5% (w/v) crystal violet for 15 min. Colonies consisting of ≥50 cells were considered positive.

### Co-immunoprecipitation and co-immunoprecipitation/Mass Spectrometry (co-IP/MS)

Cell lysates were prepared using ice-cold lysis buffer containing 0.5% NP-40, 0.25% sodium deoxycholate, 50 mM Tris-HCl (pH 7.4), 150 mM NaCl, a protease inhibitor cocktail, and phosphatase inhibitors. Lysates were pre-cleared with protein A/G agarose beads (Cat # sc-2003, Santa Cruz Biotechnology) for 1 h at 4 °C. For standard immunoprecipitation, pre-cleared lysates were incubated with 1-2 μg of primary antibodies overnight at 4 °C, followed by incubation with 20 μL of protein A/G beads for 2 h. For Flag-tagged proteins, lysates were incubated directly with anti-Flag magnetic beads (Cat # B23101, Selleck Chem, TX, USA) for 4 h at 4 °C. The beads were then washed three times with lysis buffer and resuspended in 1× SDS loading buffer. Immunoprecipitated proteins were separated by SDS-PAGE and visualized using a silver staining kit (Sangon Biotech, Cat # C500021, Shanghai, China). Approximately 1 cm^2^ of sliver-stained gel was excised, subjected to in-gel tryptic digestion, and the resulting peptides were dried before MS analysis on a Thermo Fisher Q Exactive. Raw files were processed with Thermo Scientific Proteome Discoverer v3.0 (Thermo Fisher). Proteins that directly interacts with SLC25A10-Iso3 were identified by mass spectrometry (MS), and subsequently validated by Co-IP followed by Western blotting.

### Immunohistochemistry (IHC) and multiplex immunohistochemical (mIHC) staining

Formalin-fixed paraffin-embedded HCC tissue sections were deparaffinized and subjected to antigen retrieval. The sections were then incubated with anti-SLC25A10 primary antibody (1:250 dilution) overnight at 4 °C, followed by incubation with biotinylated secondary IgG for 30 min at 37 °C. For IHC, immunoreactivity was visualized using 3,3′-diaminobenzidine (DAB) chromogen substrate, and the sections were counterstained with hematoxylin. For mIHC, tumor tissue sections (5 μm) were labeled with primary antibodies against SLC25A10-Iso3, BLC2A1, and Cleaved caspase3, followed by HRP-secondary antibodies. Subsequently, the fluorophore-conjugated tyramide amplification system (Cat # RK05903, ABclonal, Hubei, China) was used for signal amplification, and DAPI was used to counterstain the nuclei. Slides were scanned with a Leica TCS SP8 X confocal microscope equipped with a 40× oil-immersion objective.

### Immunofluorescence (IF) staining

Cells were seeded on glass coverslips in 6-well plates at a density of 1 × 10^5^ cells per well and cultured for 48 h. After washing with PBS, the cells were fixed with 4% paraformaldehyde for 30 min at room temperature, permeabilized with 0.4% Triton X-100 in PBS for 15 min, and blocked with 5% bovine serum albumin (BSA) in 0.1% Triton X-100/PBS for 30 min. Cells were then incubated overnight at 4 °C with anti-SLC25A10 antibody (1:50; Cat # WG-03144D, Abclonal, Hubei, China), followed by incubation with the appropriate fluorescent secondary antibodies and counterstaining with DAPI. Fluorescence images were captured using a Leica TCS SP8 X confocal microscope equipped with a 40× oil-immersion objective.

### Flow cytometry assay

Cells were harvested from 6-well plates, washed with PBS, and stained using an Annexin V-APC/PI apoptosis detection kit (Cat # 70-AP107-100, Multisciences, Zhejiang, China), following the manufacturer’s instructions. Approximately 1 × 10⁴ cells per sample were analyzed using a CytoFlex S flow cytometer (Beckman Coulter). The cell populations were gated based on forward scatter (FSC) and side scatter (SSC) parameters, with appropriate compensation controls applied for multicolor analysis.

### RNA-sequencing (RNA-seq) analysis

Total RNA was extracted and subjected to BGI Genomics for sequencing. The mRNA was enriched using oligo (dT) magnetic beads and fragmented for cDNA library preparation. Libraries were constructed using the NEBNext Ultra RNA Library Prep Kit (New England Biolabs) and sequenced on an Illumina HiSeq X Ten platform, generating 150 bp paired-end reads. Quality control of the raw data was performed using FastQC, and reads were aligned to the human reference genome (GRCh38) using HISAT2.

### ChIP-sequencing analysis

Chromatin immunoprecipitation sequencing (ChIP-seq) was performed using HepG2 cells fixed with 1% formaldehyde. Chromatin was prepared and immunoprecipitated using the SimpleChIP^®^ Enzymatic Chromatin IP Kit (Cat # 9003, CST, MA, USA). Purified DNA was used for library construction and was sequenced on an Illumina platform. Peak calling and motif analyses were performed using MACS2 and the MEME suite, respectively.

### Luciferase reporter assay

We obtained the promoter fragment of BCL2A1 and cloned the fragment of the BCL2A1 promoter into the pGL4.0 basic firefly luciferase reportervector using restriction endonuclease. The reporter vector was transfected into Huh7 overexpressing SLC25A10 isforms using Lipofectamine 2000. After 48 h of incubation, luciferase reporter gene activity was measured using the Dual Luciferase^®^ Reporter Assay System (Cat # E1910, Promega, WI, USA).

### Measurements of cellular ATP levels

Cellular ATP levels were determined in macrophages by an ATP Assay Kit (Cat # S0027, Beyotime, Jiangsu, China) following manufacturer’s protocol. Huh7 cells were treated with Diethyl butylmalonate (8 mM) for 24 h, washed and harvested after mixing with nucleotide releasing buffer and incubated for 5 min at room temperature with gentle shaking. Then, 100 μL prepared reaction mix was added in control wells and the background luminescence was read (Data A), then 20 μL sample was added and after 2 min the luminescence was read (Data B). The data (Data B −Data A) is to consume background. Calculate the ATP concentration in the sample based on the standard curve.

### Clinical specimens

Primary liver cancer specimens were obtained from Ruijin Hospital, Shanghai Jiao Tong University School of Medicine, with approval from the Institutional Review Board (IRB approval no. 2020 (172)). Written informed consent was obtained from all patients prior to sample collection. The inclusion criteria were as follows: (1) histologically confirmed diagnosis of HCC, (2) no prior neoadjuvant therapy, and (3) availability of complete clinical follow-up data. Specimens were either snap-frozen in liquid nitrogen or formalin-fixed and paraffin-embedded for subsequent analysis.

### Xenograft mouse model

Five- to six-week-old male athymic nude mice (Ncr-nu/nu; SLAC Laboratory Animal Co., Shanghai, China) were maintained under specific pathogen-free conditions. For tumor implantation, 5 × 10⁶ Huh7 cells suspended in 100 μL of a 1:1 mixture of PBS and Matrigel were injected subcutaneously into the right flank. Tumor dimensions were measured twice weekly with calipers, and the volume was calculated using the following formula: (length × width²)/2. When the tumor volume reaches 200 mm^3^, the mice bearing tumors were randomly grouped (with five to six mice in each group), and Etoposide treatment was administered via intraperitoneal injection every other day (at a dose of 20 mg/kg). Animals are euthanized under isoflurane anesthesia by cervical dislocation and the tumors are weighed when the tumor volume exceeds 2000 mm³, body weight loss exceeds 15%, or severe complications appear. All procedures were approved by the Institutional Animal Care and Use Committee of the Shanghai Jiao Tong University (IACUC protocol no. JUMC2023-023-A) and conducted in accordance with the AAALAC guidelines.

### Statistical analysis

Data are presented as the mean ± standard deviation (SD). Statistical significance between two groups was determined using unpaired two-tailed Student’s *t* tests, while comparisons involving multiple groups were analyzed using one-way ANOVA followed by Tukey’s post-hoc test. Statistical significance was set at *P* < 0.05. All experiments were independently repeated at least three times, with consistent results. Statistical analyses were performed using the GraphPad Prism version 9.0.

## Supplementary information

Supplemental figures

Uncrossed Western blots

Supplementary figure S5

## Data Availability

All other data were obtained from the corresponding author upon reasonable request. The RNA sequences have been deposited in NCBI under accession numbers GSE279928 and GSE279929.
