# Supplementary material for: Nuclear translocation of SLC25A10 isoform 3 promotes chemoresistance in HCC cells via CEBPB/BCL2A1 signaling
Source: Cell Death Dis. 2026 Apr 9;17(1):491. doi: 10.1038/s41419-026-08667-4 (PMC13187160; doi:10.1038/s41419-026-08667-4)
Supplement: Supplementary file 1 — Supplemental figures [file 41419_2026_8667_MOESM1_ESM.pdf]

Figure S1

A

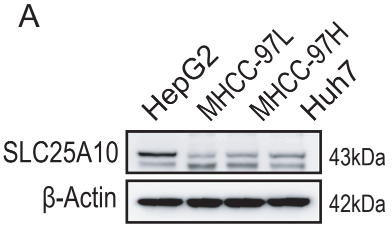

Figure legend

A. Expression of SLC25A10 iso 3 in various HCC cell lines.

Figure S2

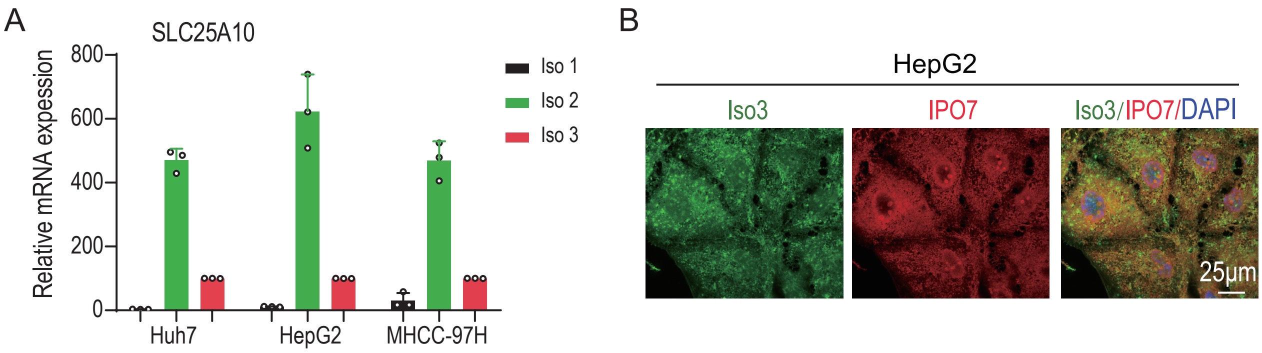

Figure S2

A. Analysis of SLC25A10 isoform expression in HCC cell lines.

B. Confocal microscopy of SLC25A10 isoform 3 and IPO7 in HepG2 cells under hypoxia .

Figure S3

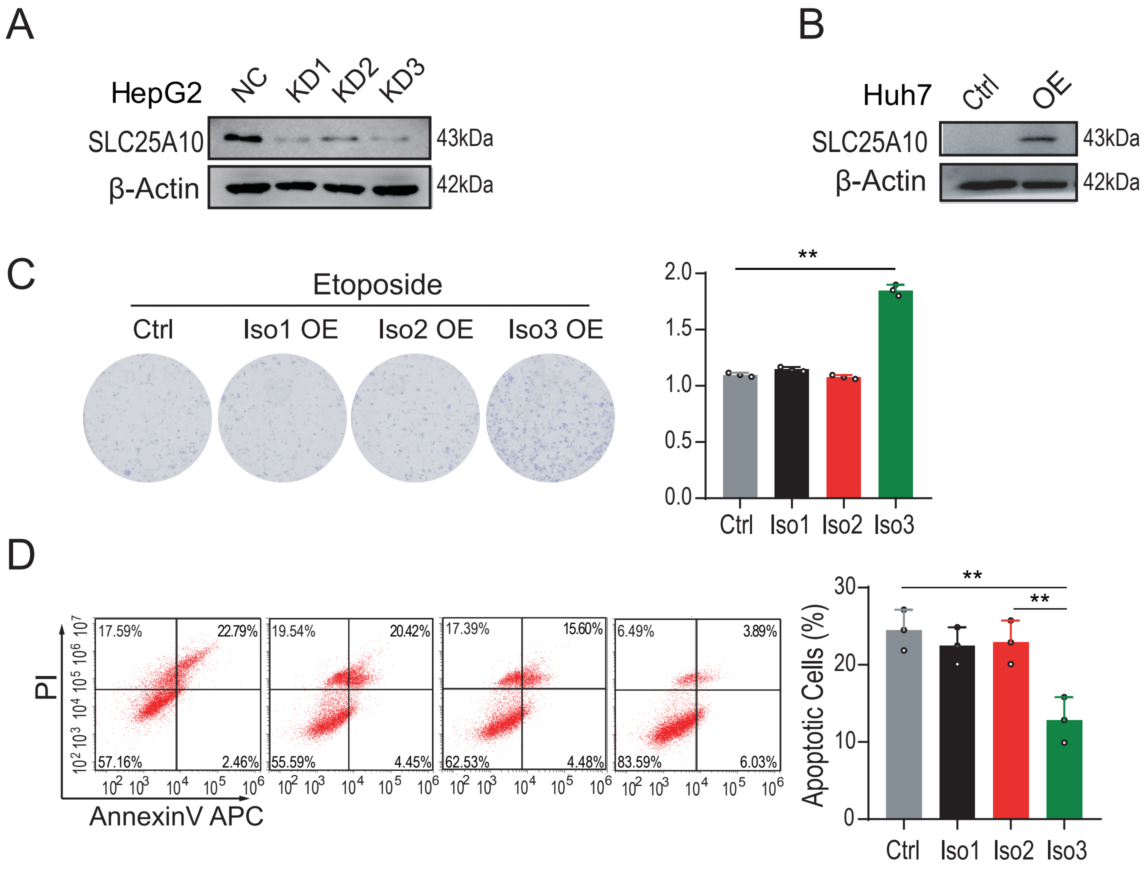

Figure legend

A. SLC25A10 iso 3 knockdown efficiency is detected in HepG2 cells, which is upregulated compared to the other HCC cells.

B. SLC25A10 iso3 overexpression efficiency is detected in Huh7 cells. Experiments were repeated independently three times.

C. Assessment of iso 1/2/3 overpexression on Etoposide-induced cell viability in Huh7 cells via colony formation assay.

D. Evaluation of iso 1/2/3 overpexression on Etoposide-induced apoptosis in Huh7 cells via flow cytometry.

Figure S4

A

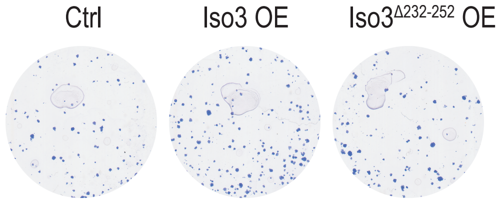

Figure legends:

A. Assessment of SLC25A10 iso3 or iso3$^{\Delta 232\text{-}252}$ impact on Huh7 cell survival following Etoposide treament via colony formation assay. Huh7 cells (10^3 cells/well) were and treated with 2.5 μM Etoposide. Colony formation was evaluated after 10 days of growth. Experiments were performed in triplicate (n = 3 per group) and repeated independently three times.

Figure S6

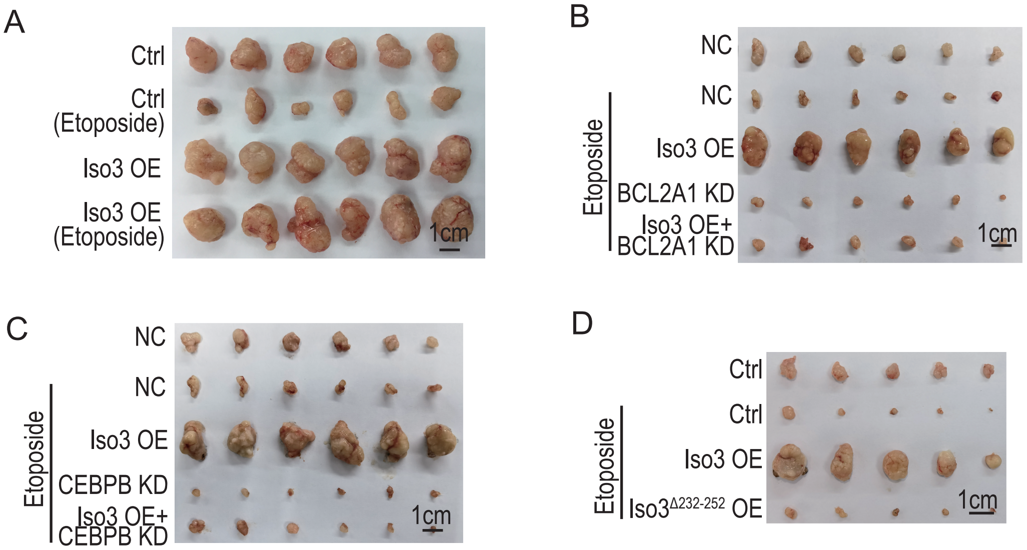

Figure legend

A-D. Representative tumor photos in each groups.
