# Supplementary figures and images for: Nuclear translocation of SLC25A10 isoform 3 promotes chemoresistance in HCC cells via CEBPB/BCL2A1 signaling
Source: Cell Death Dis. 2026 Apr 9;17(1):491. doi: 10.1038/s41419-026-08667-4 (PMC13187160; doi:10.1038/s41419-026-08667-4)

Figure 1

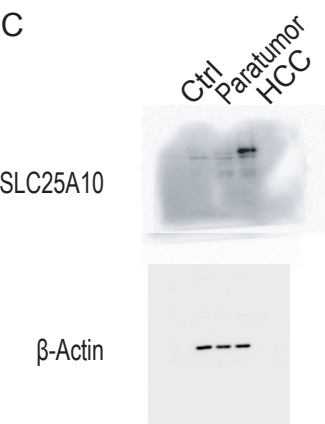

Figure 2

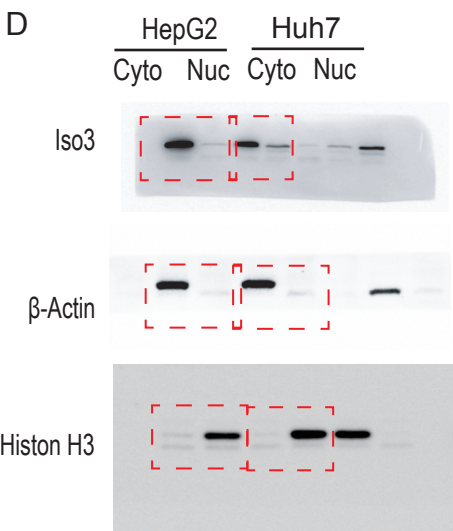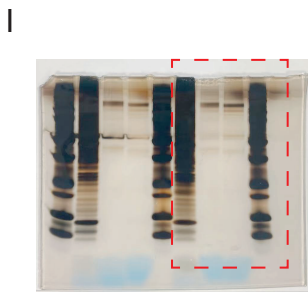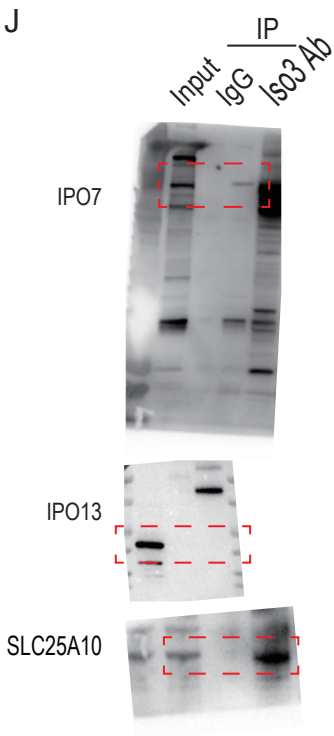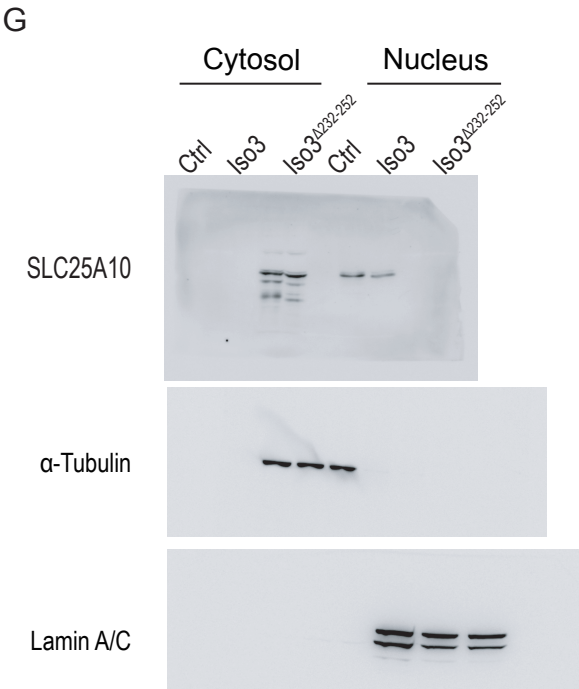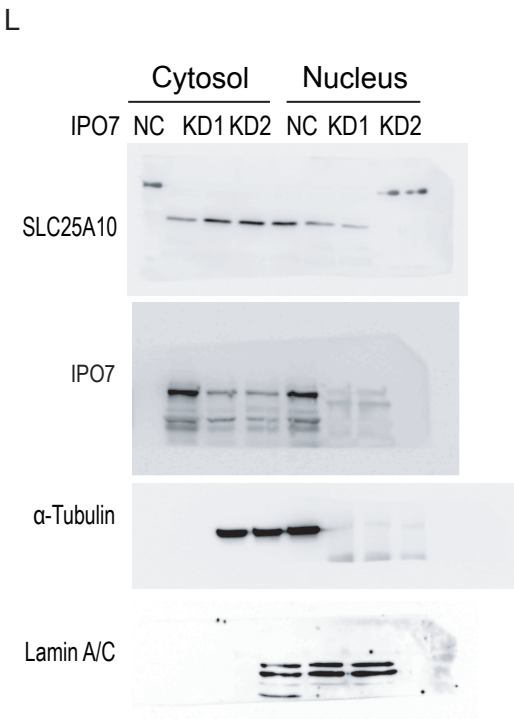

Figure 3

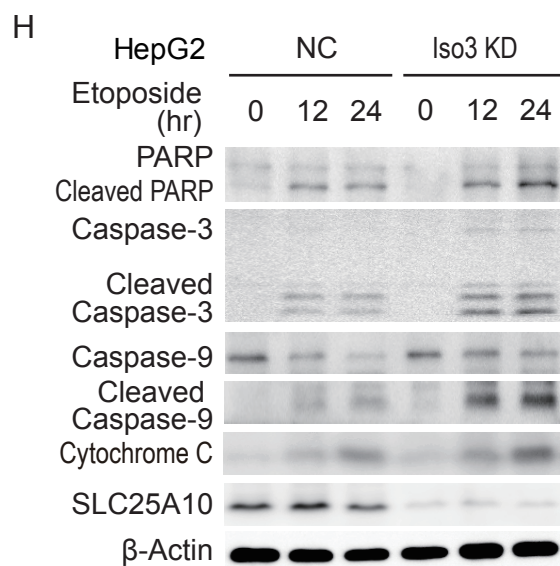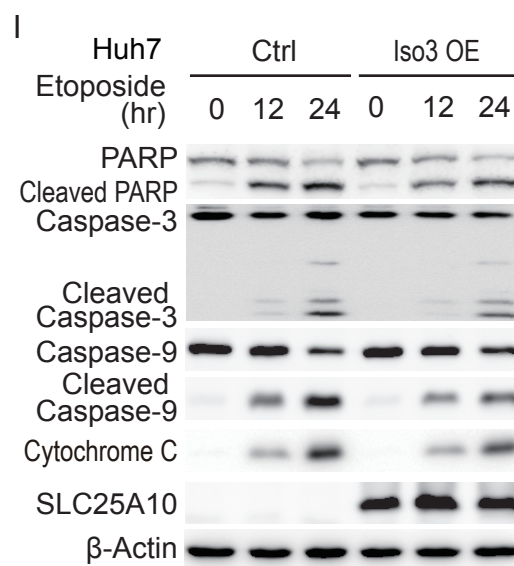

Figure 4

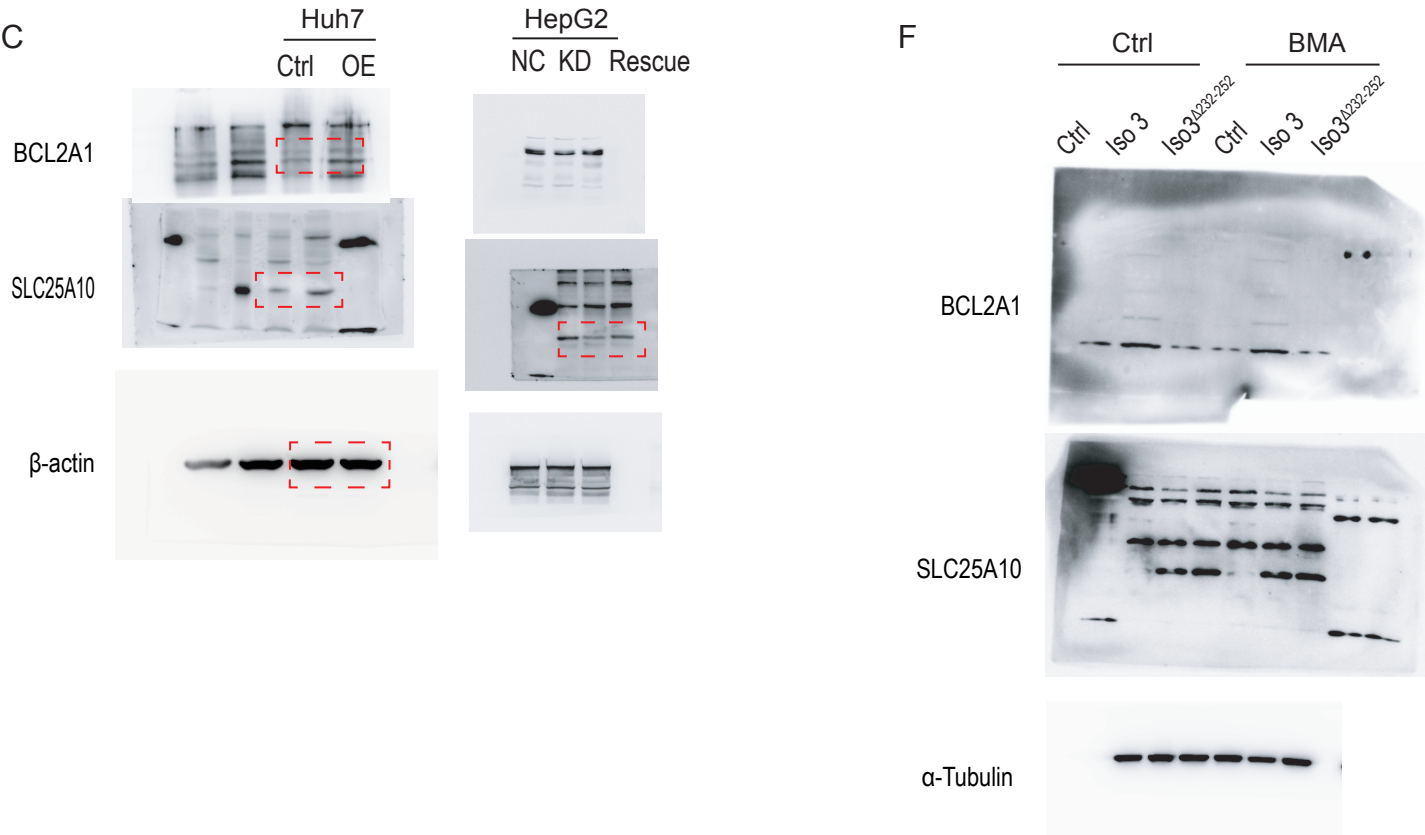

Figure 5

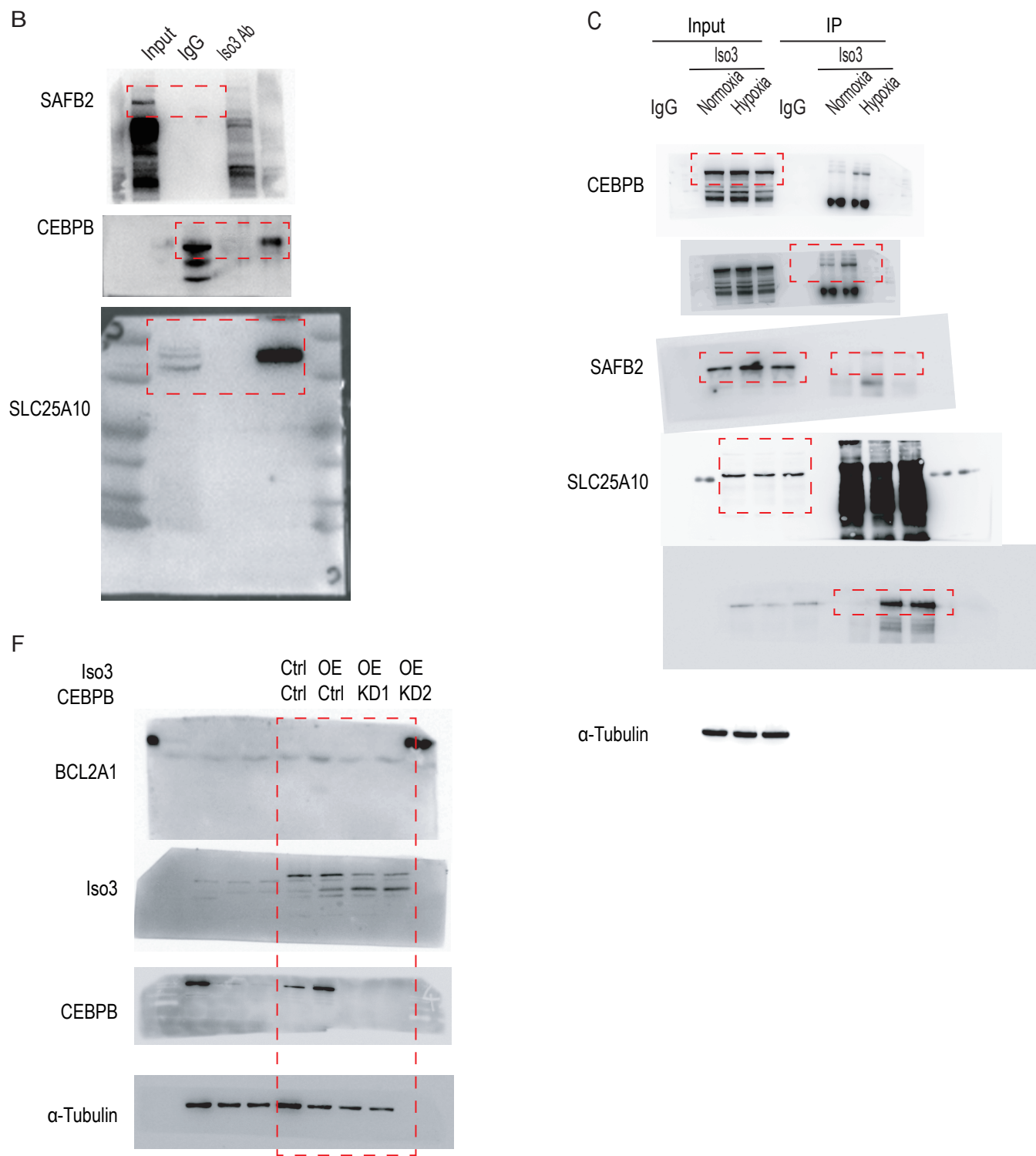

Figure 6

B

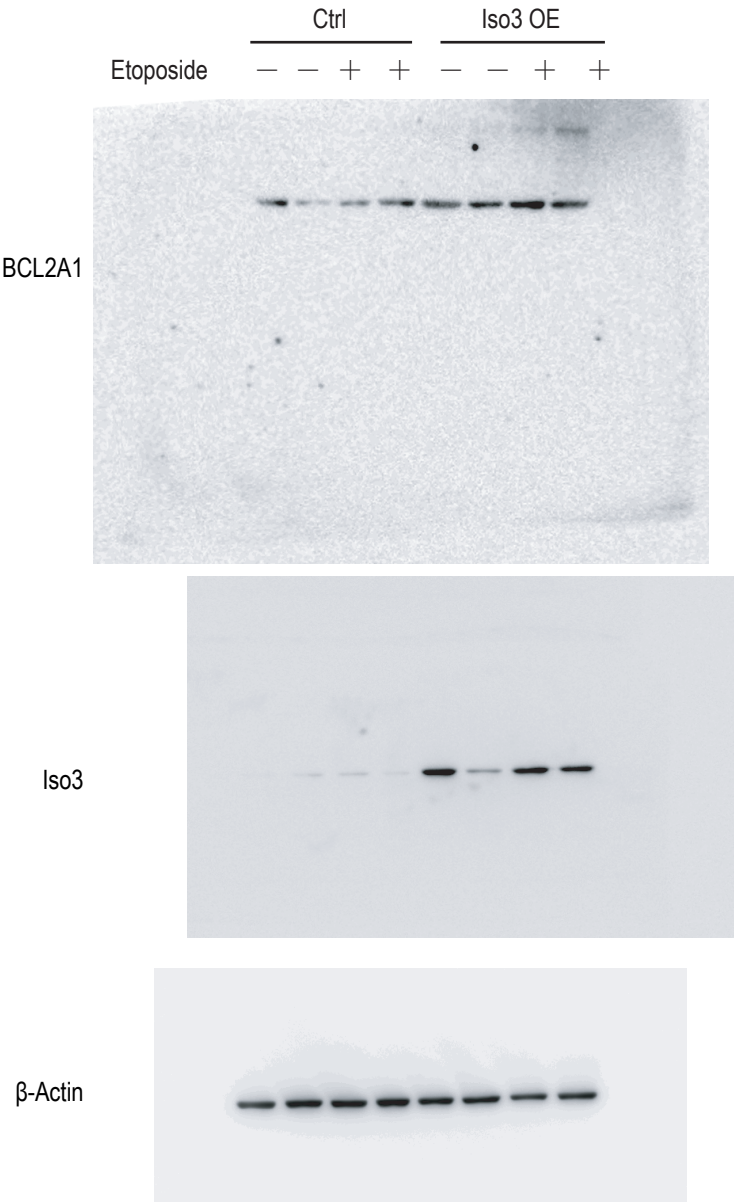

Figure S1

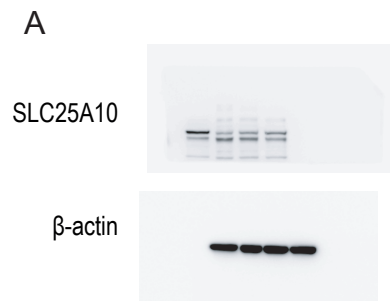

Figure S3

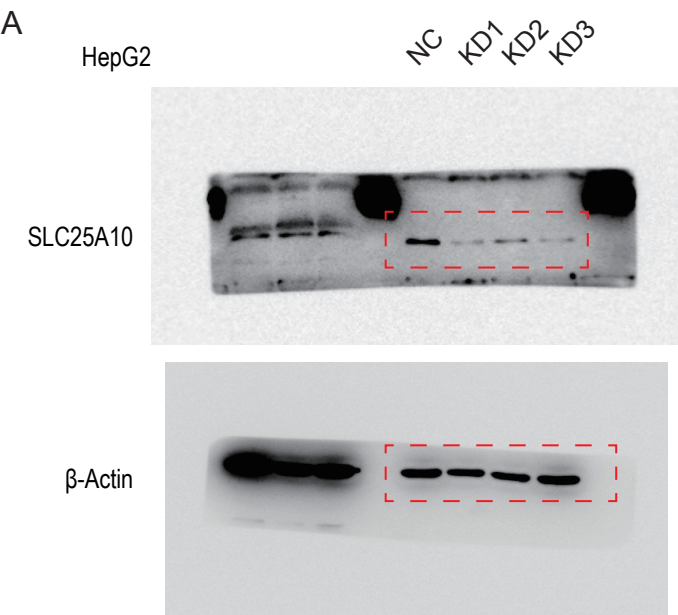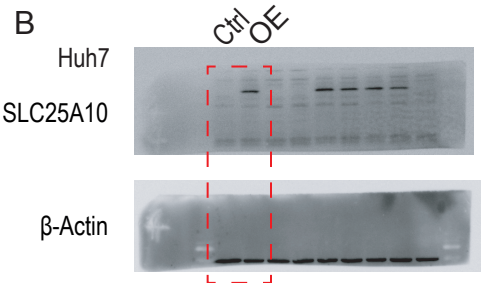

Supplement: Supplementary file 2 — Uncrossed Western blots [file 41419_2026_8667_MOESM2_ESM.pdf]
