# Supplementary material for: Nuclear translocation of SLC25A10 isoform 3 promotes chemoresistance in HCC cells via CEBPB/BCL2A1 signaling
Source: Cell Death Dis. 2026 Apr 9;17(1):491. doi: 10.1038/s41419-026-08667-4 (PMC13187160; doi:10.1038/s41419-026-08667-4)
Supplement: Supplementary file 3 — Supplementary figure S5 [file 41419_2026_8667_MOESM3_ESM.pdf]

Figure S5

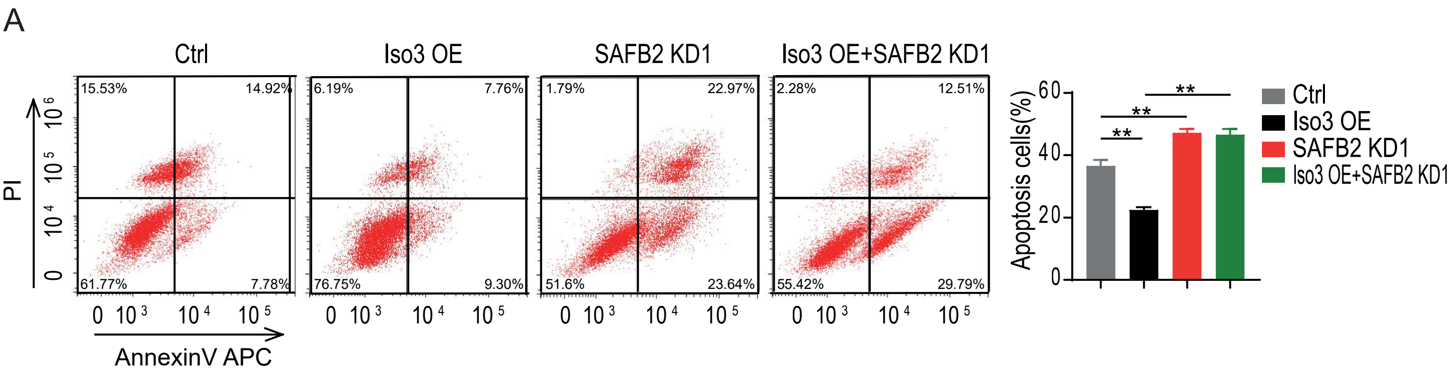

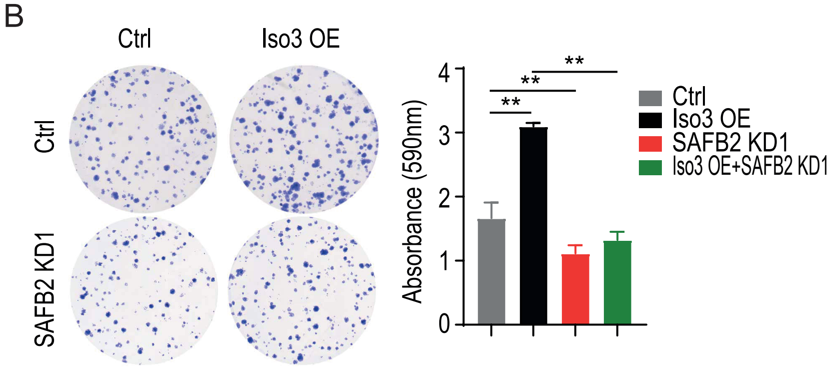

Figure legends:

A. Apoptosis was evaluated by flow cytometry following SAFB2 knockdown in Huh7 cells treated with 15 μM Etoposide for 24 hours. Experiments were repeated independently three times.

B. Assessment of SAFB2 knockdown effect on Huh7 cell survival via colony formation assay. Cells (10^3 cells/well) were treated with 2.5 μM Etoposide for 10 days, and colony formation was quantified. Assays were performed in triplicate (n = 3 per group) with three independent replicates.
